# Lipid nanoparticle-based mRNA vaccines: a new frontier in precision oncology

**DOI:** 10.1093/pcmedi/pbae017

**Published:** 2024-08-01

**Authors:** Eden M Jacob, Jiaoti Huang, Ming Chen

**Affiliations:** Department of Pathology, Duke University School of Medicine, Durham, NC 27710, USA; Duke Cancer Institute, Duke University, Durham, NC 27710, USA; Department of Pathology, Duke University School of Medicine, Durham, NC 27710, USA; Duke Cancer Institute, Duke University, Durham, NC 27710, USA; Department of Pathology, Duke University School of Medicine, Durham, NC 27710, USA; Duke Cancer Institute, Duke University, Durham, NC 27710, USA

**Keywords:** lipid nanoparticles, mRNA vaccines, cancer

## Abstract

The delivery of lipid nanoparticle (LNP)-based mRNA therapeutics has captured the attention of the vaccine research community as an innovative and versatile tool for treating a variety of human malignancies. mRNA vaccines are now in the limelight as an alternative to conventional vaccines owing to their high precision, low-cost, rapid manufacture, and superior safety profile. Multiple mRNA vaccine platforms have been developed to target several types of cancer, and many have demonstrated encouraging results in animal models and human trials. The effectiveness of these new mRNA vaccines depends on the efficacy and stability of the antigen(s) of interest generated and the reliability of their delivery to antigen-presenting cells (APCs), especially dendritic cells (DCs). In this review, we provide a detailed overview of mRNA vaccines and their delivery strategies and consider future directions and challenges in advancing and expanding this promising vaccine platform to widespread therapeutic use against cancer.

## Introduction

**Figure 1.**
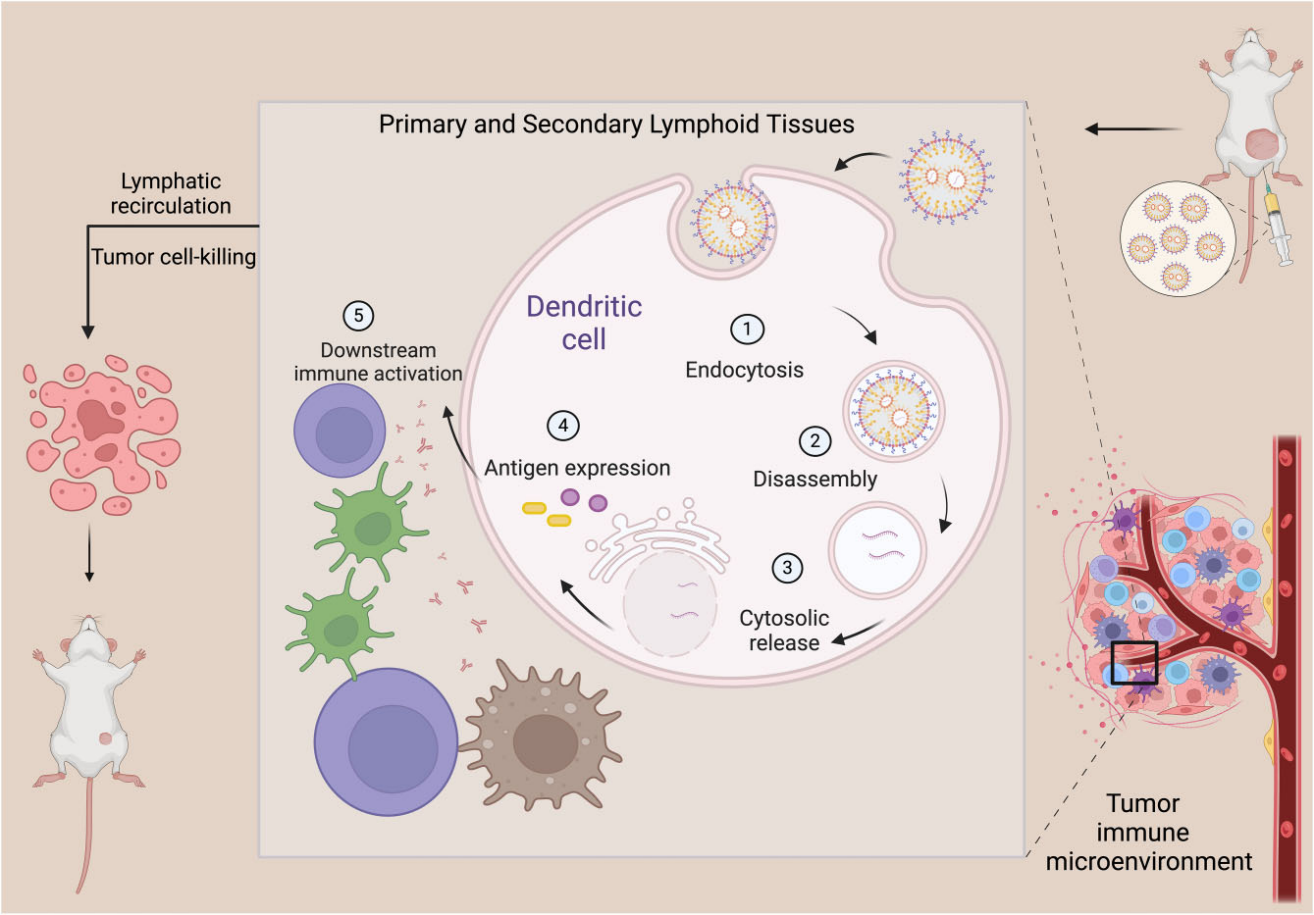
Diagram illustrating the *in vivo* administration of lipid nanoparticle-encapsulated mRNA vaccine into tumor models, its internalization by dendritic cells, downstream immune activation, effector T cells homing from lymphoid tissues to the tumor sites through lymphatic recirculation, and subsequent tumor cell-killing.

Cancer immunotherapies reshape the tumor immune microenvironment and drive the activation of host anti-tumor immunity to suppress tumorigenesis and growth. Cancer vaccines thus represent a promising strategy for achieving this specialized anti-tumor immunotherapy as they can mobilize T cell responses against tumor-associated antigens (TAAs) or tumor-specific antigens (TSAs) to specifically attack and destroy malignant tumor cells (Fig. 1) [1]. The high expression of antigens induced by vaccines can sustain organismal tumor-killing ability through immune memory. Thus, cancer vaccines could theoretically provide therapy that is more precise, safer, better-tolerated, and longer-lasting than other approaches (Fig. 1).

After its discovery in 1961 [2], mRNA was soon recognized as a potential therapeutic delivery system and in the 1970s nucleic acid-based vaccine solutions became the subject of intense drug research [3]. After years of effort, and as medical technology progressed, preclinical studies of mRNA-based vaccines and cancer immunotherapies began to show improved rates of success [4]. In 1990, the first successful expression of *in vitro* transcription (IVT) of mRNA in mouse skeletal muscle cells through direct injection into animals demonstrated the feasibility of mRNA vaccine development [5]. Since then, mRNA has been widely credited with the potential to revolutionize vaccination, cancer immunotherapies, cellular reprogramming, and genome editing [6]. On the other hand, although manipulating protein expression may ultimately prove a powerful weapon in conquering diseases, proteins have been limited as therapeutic agents by their large size and high cost of production, and research has therefore focused on exogenously produced nucleic acids and their introduction into cells. Plasmid DNA was initially pursued as a therapeutic vector [7], however, IVT of mRNA had various advantages in therapeutic applications, including low toxicity and high translatability in both dividing and non-dividing cells, as RNA only needs to be internalized into the cytoplasm (rather than the nucleus), where a one-step translation can produce the antigen(s) of interest and initiate immunostimulatory activity for cancer immunotherapy [7]. mRNA vaccines have higher rates and greater magnitude of protein expression than DNA vaccines due to the relatively high transfection efficacy of mRNA. Additionally, mRNA cannot integrate into the genome sequence, precluding any risk of insertional mutagenesis. Moreover, synthesizing mRNA in a cell-free system in suitable standardized and controlled conditions makes production of good manufacturing practice-grade mRNA comparatively simple, rapid, and inexpensive at a range of scales [8]. These attributes gave mRNA vaccines critical advantages in response to the coronavirus pandemic, and US Food and Drug Administration (FDA) approval of two mRNA-based vaccines from Pfizer-BioNTech and Moderna for emergency use against COVID-19 led to a dramatic rise in the mRNA-based pharmaceutical market and development of applications of mRNA therapies against both cancer and infectious disease [9].

Direct injection of naked mRNA into the body, however, faces several hurdles in clinical application, including its quick degradation via endonucleases, the obstruction of cell internalization due to dense negative charges of nucleic acids, and the triggering of nonspecific interferon responses [10]. Remedies such as adding effective shielding from degradation and enhancing gene translation in the cell became necessary to ensure the efficacy of vaccine delivery. In addition, a nonviral vector delivery system was developed that utilized lipid nanoparticles (LNPs) as carriers due to their nanoscopic size (diameter < 200 nm), biocompatibility, safety, and ease of scalability [11]. These nano-delivery systems have now become widely favored for the delivery of anti-inflammatory, antioxidant, and anti-cancer agents due to their many advantages over conventional medicines [8]. Specifically, the encapsulation of mRNA within LNPs improves stability and mediates controlled, targeted delivery of the therapeutics to diseased cells [12].

**Figure 2.**
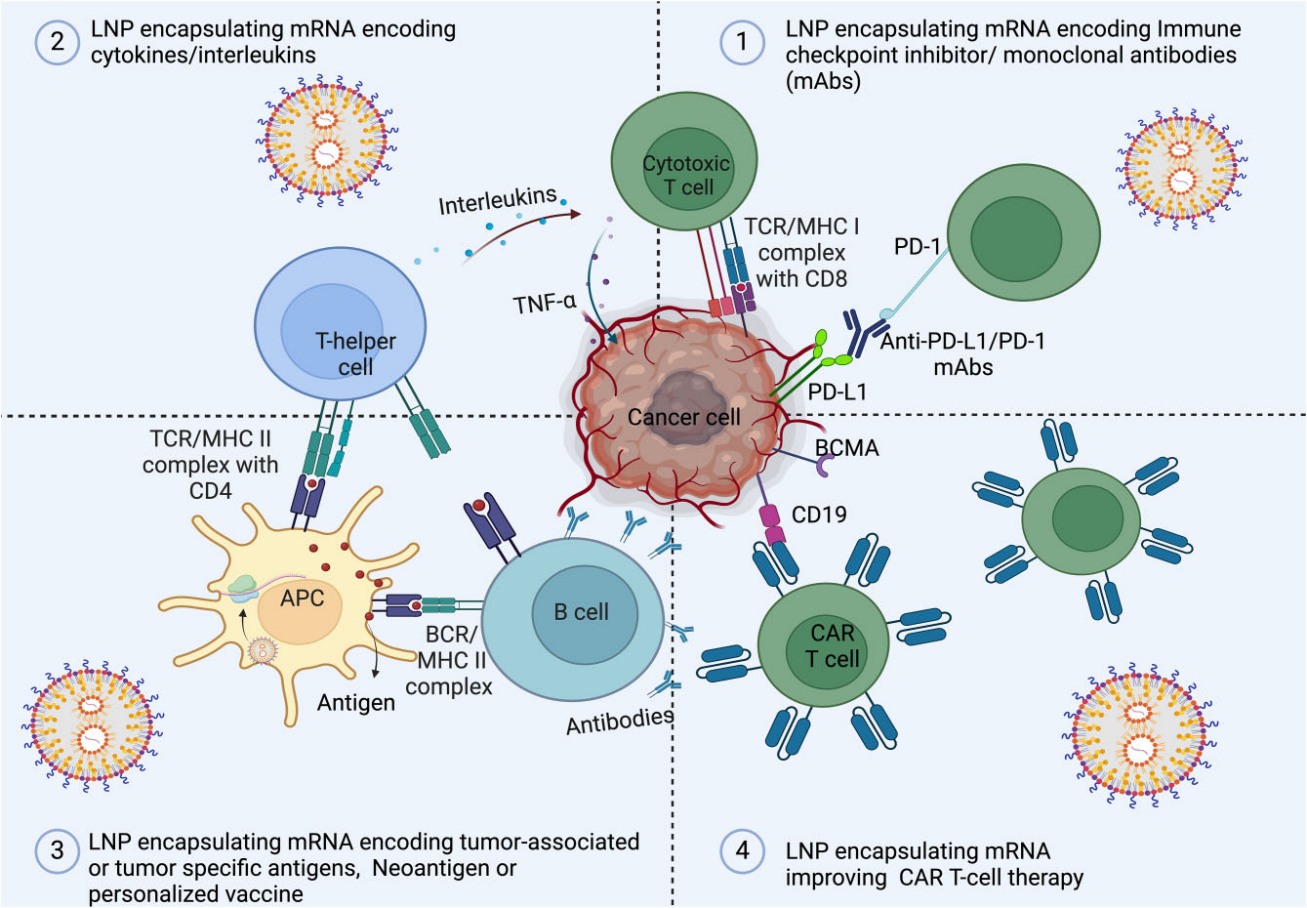
Schematic diagram of different mechanisms of LNP mRNA vaccines in cancer treatment. APC: Antigen presenting cells; BCR: B-cell receptor; MHC: major histocompatibility complex; PD-1: programmed cell death protein-1; PD-L1: programmed death ligand-1; TCR: T-cell receptor; TNF-α: tumor necrosis factor alpha.

In this article we will provide an overview of current advances and challenges in different LNP-based mRNA vaccines for cancer immunotherapies, including vaccines that encode (i) monoclonal antibodies (mAbs), (ii) interleukins (ILs) and cytokines, (iii) TAAs, TSAs, and neoantigens, or that improve (iv) chimeric antigen receptor (CAR) T-cell therapy (Fig. 2).

## LNP aiding mRNA delivery

**Figure 3.**
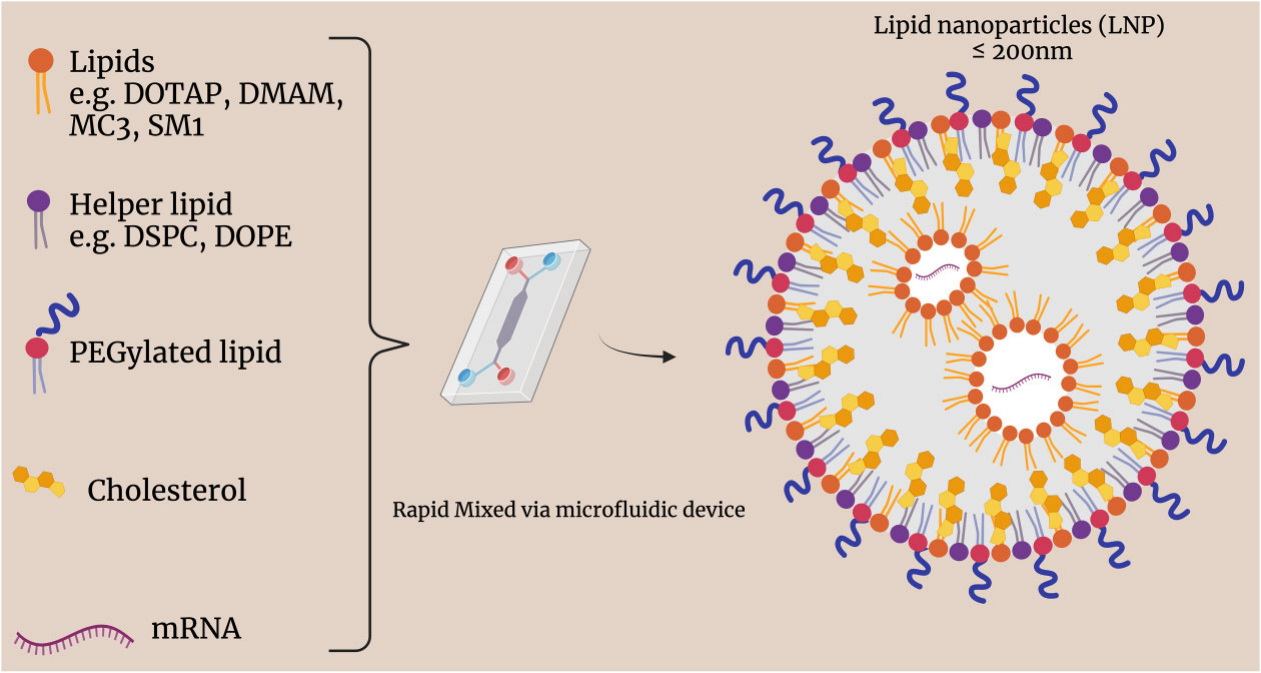
Illustration of synthesis of LNP-encapsulated mRNA vaccine material for cancer treatment.

Currently the most well-developed tools for mRNA delivery depend on lipid-based systems. A cationic lipid not only aids in encapsulating the polyanionic mRNA but also interacts with negatively charged phospholipids in the plasma membrane to stimulate internalization by endocytosis [8]. The typical constituents of an LNP (Fig. 3) are a combination of four key elements: first, for the encapsulation of the polyanionic mRNA, a pH-responsive or cationic lipid bearing tertiary or quaternary amine is essential; second, a neutral helper lipid, such as 1,2-dioleoyl-*sn*-glycero-3-phosphoethanolamine (DOPE) or 1,2-distearoyl-*sn*-glycero-3-phosphocholine (DSPC), maintains the bilayer structure and aids cellular uptake; third, a sterol lipid-like cholesterol stabilizes the lipid bilayer of LNPs and promotes membrane fusion, thereby boosting the efficiency of mRNA delivery; and finally, the addition of polyethylene glycol (PEG)-lipid can reduce aggregation and establish a hydration layer over the LNPs that decreases non-specific uptake and prevents the absorption of plasma proteins and avoids reticuloendothelial clearance, thus enhancing colloidal stability [13, 14].

Kranz *et al*. [15] demonstrated that by optimizing the mRNA/cationic lipid ratio and using *N*-[1-(2,3-dioleyloxy)propyl]-*N,N,N*-trimethylammonium chloride (DOTMA)/DOPE or 1,2-dioleoyl-3-trimethylammonium-propane (DOTAP)/DOPE formulations, dendritic cells (DCs) could be passively targeted by solely adjusting the negative net charge of the nanoparticles; no molecular ligand functionalization was required. Delivery of the mRNA was safeguarded via a lipoplex nanostructure that minimized the degradation inflicted by extracellular ribonucleases. The internalization of the LNPs in various lymphoid organs induced translation from the encapsulated mRNA in a range of DC subsets and macrophages. The mRNA-lipoplex vaccine also stimulated both an innate type-I interferon (IFN)-mediated immune response and a potent adaptive response, which together led to a strong rejection of aggressive tumors [15]. One substantial obstacle to the delivery of mRNA vaccines for cancer immunotherapy, i.e. inadequate accumulation in antigen-presenting cells (APCs), was successfully addressed by a recent study. By exploiting mannose receptor-mediated endocytosis, Lei *et al*. [16] designed DC-targeting LNPs (STLNPs-Man) for mRNA delivery *in vitro* and *in vivo*. Compared to vaccines based on commercially available LNPs, the mRNA vaccine (STLNPs-Man@mRNAOVA) showed a 4-fold increase in DC uptake when administered intramuscularly. STLNPs-Man@mRNAOVA necessitated only one-fifth the dose of commercial LNPs, thus reducing the likelihood of adverse effects. Additionally, STLNPs-Man@mRNAOVA inhibited the CD206/CD45 axis, resulting in downregulation of cytotoxic T-lymphocyte-associated protein 4 (CTLA-4) on T cells. This implies substantial potential for improved efficacy when used in conjunction with immune checkpoint inhibitors (CPIs), and this method is therefore a promising advance in the design of mRNA vaccines for cancer treatment, as it offers significantly reduced toxicity as well as improved efficacy [16]. Additionally, the incorporation of immune adjuvant α-galactosylceramide along with the mRNA encapsulated in lipopolyplex nanoparticles enabled passive targeting of DCs and induced a substantial therapeutic effect against a highly malignant B16-F10 melanoma tumor [17]. Moreover, mRNA lipopolyplexes with mannose receptor-targeting moieties did not depend on type-I IFN for effective T-cell immunity. This differential anti-tumor T-cell immunity of mRNA lipopolyplexes enabled the incorporation of N1 methyl pseudouridine nucleoside-modified mRNA to generate mRNA vaccines with potent immunogenicity yet low and safe inflammatory responses [18], which could provide a powerful therapeutic alternative to mRNA-lipoplex vaccines currently being evaluated in early phase clinical trials.

## Application of LNP-based mRNA cancer vaccines

The resurrection of mRNA cancer vaccines as a general therapeutic application can be attributed to the swift development of RNA-based vaccines during the COVID-19 pandemic and their success against SARS-COV-2 [19]. mRNA has a brief half-life in the blood and is hampered from entering target cells [20]. A wide variety of LNPs, including lipids, lipid derivatives, and hybrid particles, have been through extensive testing and have been effectively implemented for clinical trials in the delivery of mRNA [8] (Table 1), and these nanomedicine techniques have transformed the management of many diseases, including cancer. The small size (1–200 nm) and large surface-to-volume ratio of LNPs, along with their tunable surface functionalization properties, offer even bio-distribution, enhanced encapsulation of drugs and nucleic acid, controlled drug release, and reduced systemic toxicities [21]. Moreover, these LNPs boast favorable pharmacokinetics and have shown potent pharmacological effects against numerous diseases [22]. Currently, various immunotherapies based on mRNAs have been applied in clinical trials, and results have confirmed the efficacy of a range of treatments against solid tumors [23]. The following section describes LNP-based mRNA cancer vaccines that are currently in preclinical or clinical trials (Table 1).

**Table 1.** Representative LNP-based mRNA vaccines in clinical trials

Vaccine/administration	Encoded protein	Condition	Adjuvant therapy	NCT number	Trial phase	Study start date	Trial outcomes
mRNA-2416/intratumorally	OX40L	Solid tumor or lymphoma	With or without Durvalumab	NCT03323398 [24]	I/II	15 August 2017	This study was halted prematurely on 18 August 2021 because the efficacy endpoints were not met for either treatment arm.
mRNA-2752/intratumorally	OX40L, IL-23, IL-36γ	Solid tumor or lymphoma	With or without Durvalumab	NCT03739931 [25, 26]	I	27 November 2018	This study is active, not recruiting, and is expected to be completed on 10 march 2026. As of 1 July 2022, mRNA-2752 alone or combined with Durvalumab was found to be safe and tolerable with preliminary efficacy in immune refractory tumors, including triple-negative breast cancer and melanoma [26].
FixVac (BNT111)/intraveously	NY-ESO-1, tyrosinase, MAGE-A3, TPTE	Advanced melanoma	With or without anti-PD1	NCT02410733 [27]	I	March 2015	The study was completed on 20 June 2023. In the mRNA vaccine monotherapy cohort: 3 patients (12%) experienced a partial response, while 7 (28%) maintained stable disease. In the mRNA vaccine combined with anti-PD1 therapy cohort: 6 patients (35%) exhibited a partial response, while 2 (12%) maintained stable disease.
CV9201/intradermally	MAGE-C1, MAGE-C2, NY-ESO-1, survivin, 5T4	Stage IIIB/IV non-small cell lung cancer	None	NCT00923312 [28]	I/IIa	May 2009	The study was completed on 20 March 2018. No objective tumor responses to CV9201 were seen; the observed progression-free survival (PFS) and overall survival are consistent with NSCLC patients not progressing after first-line chemotherapy.
CV9202/intradermally	MAGE-C1, MAGE-C2, NY-ESO-1, survivin, 5T4, MUC1	Stage IV non-small cell lung cancer	With local radiation	NCT01915524 [29, 30]	Ib	April 2013	The study was terminated in July 2016 because of inadequate enrollment within the anticipated timeframe. One patient (4%) achieved a partial response in combination with pemetrexed maintenance, while 12 (46%) maintained stable disease.
CV9202/intradermally	MAGE-C1, MAGE-C2, NY-ESO-1, survivin, 5T4, MUC1	Metastatic non-small cell lung cancer	With Durvalumab (arm A) or with Durvalumab + Tremelimumab (arm B)	NCT03164772 [31]	I/II	20 December 2017	The study was completed on 29 October 2021. The results showed that at the eighth week after treatment, the PFS rate was 47.8 and 32.4% in the arm A and arm B cohorts, respectively. At the 24th week after treatment, the PFS rate was 43.5% in arm A and 8.8% in arm B.
mRNA-4157 (V940)/intramuscularly	Up to 34 personalized neoantigens	Resected solid tumors	With or without Pembrolizumab	NCT03313778 [32]	I	14 August 2017	The study will be completed on 30 June 2025. As of 10 May 2019, no dose limiting toxicities or related serious adverse events or adverse events ≥ grade 3 were reported. Neoantigen-specific T cell responses were detected, supporting the advancement of mRNA-4157 to Phase II.
mRNA-4157 (V940)/intramuscularly	Up to 34 personalized neoantigens	Stage III/IV melanoma	With Pembrolizumab	NCT03897881 [33, 34]	II	18 July 2019	Recruitment expected to be completed by 09 September 2029. Based on earlier positive data showing superior recurrence-free survival in high-risk melanoma patients treated with mRNA-4157 in combination with Pembrolizumab versus Pembrolizumab [33], the US FDA and European Medicine Agency granted Breakthrough Therapy Designation on 22 February 2023 [35] and Prime Scheme Designation on 6 April 2023 [36], respectively, for mRNA-4157 in combination with Pembrolizumab for the adjuvant treatment of patients with high-risk melanoma following complete resection. Multiple Phase III trials (NCT06077760, NCT06295809, NCT05933577) were also initiated.
RO7198457 (BNT122)/intravenously	Up to 20 personalized neoantigens	Stage III/IV colorectal cancer	None	NCT04486378 [37]	II	8 March 2021	This study is recruiting and has no results posted. It is expected to be completed by February 2026.

### mRNA cancer vaccines encoding mAbs

mAbs have been exploited for clinical use since they were first successfully produced in the laboratory through immortalizing B cells mimicking the naturally produced antibodies within the body that target specific antigens [38]. mAbs have revolutionized clinical approaches to therapy as they are precise, effective, and result in reduced side effects, especially in treating certain types of cancer [38]. Immune checkpoint mAbs such as anti-programmed cell death 1 (PD-1)/programmed cell death-ligand 1 (PD-L1) and CTLA-4 have shown durable responses in the treatment of melanoma, non-small cell lung cancer (NSCLC), and renal cancer carcinoma [39]. Although recombinant technologies have expanded the utilization of this quickly growing class of therapeutics, the utilization of these recombinant antibodies required improvements in such aspects as the formulation features that suppress aggregation during long-term storage, broader biodistribution, the necessity for large-scale manufacturing, the need for complex protein characterization, and the high costs of repeated administration during treatment [38, 40]. Since mRNAs encode proteins, full-size mAbs, antibody fragments, or any antibody variants can theoretically be coded and delivered to cells, which subsequently produce these proteins. Pardi *et al*. [41] first demonstrated the feasibility of using mRNA to encode the light and heavy chains of VRC01, an antibody against HIV-1. Thran *et al*. [42] independently validated the feasibility of using mRNA for passive vaccination against infectious agents, toxins, and tumors. Their findings demonstrated that single injections of mRNA-LNPs were sufficient to establish rapid, strong, and long-lasting serum antibody titers *in vivo*, and that therapeutic mRNA-mediated antibody expression allowed mice to survive an otherwise lethal tumor challenge. An optimized IVT-mRNA system for *in vivo* delivery of a humanized anti-Her2 antibody, Trastuzumab, was developed by Rybakova *et al*. [43]. Systemic delivery of optimized IVT-mRNA loaded into LNPs improved the pharmacokinetic profile for *in vivo* produced Trastuzumab compared to injected Trastuzumab protein, and resulted in selective anti-tumor activity in HER2-positive tumors while improving animal survival. Wu *et al*. [44]. designed the IVT-mRNA encoding Pembrolizumab, a commercially available anti-PD-1 mAb. Maximized Pembrolizumab expression levels from IVT-mRNA were achieved by optimizing the signal peptide and the molar ratio of the heavy/light chain. A single dose of IVT-mRNA loaded into LNPs in mice resulted in serum Pembrolizumab levels that endured for >35 days and displayed a significantly enhanced pharmacokinetic profile compared to the same dose of directly injected Pembrolizumab. Chronic treatment of tumor-bearing mice with LNP-encapsulated Pembrolizumab mRNA effectively suppressed the growth of intestinal tumors and improved animal survival. These studies indicate that mRNA‐encoding antibodies appear to represent a viable therapeutic strategy against various biological threats, including (viral) infections, intoxication, and cancer [41–44].

Furthermore, other research has yielded an innovative approach to mRNA delivery with bispecific T-cell engaging (BiTE) antibodies. The therapeutic potential of BiTE antibodies has been hindered by manufacturing challenges and their short serum half-life [45]. Stadler *et al*. [46]. hypothesized that these limitations could be circumvented by generating BiTE antibodies using mRNA. RiboMABs, BiTE antibodies directed against the T cell receptor-associated molecule CD3 and one of three TAAs (CLDN6, CLDN18.2, or EpCAM), were generated *in vivo* using 1-methylpseudouridine-containing mRNAs. RiboMAB levels were sustained over several days *in vivo* and their therapeutic efficacy against advanced xenograft tumors was equivalent to that of recombinant antibodies. Additionally, mRNA encoding B7H3 × CD3 BiTE encapsulated in LNPs have achieved high transfection efficiency and targeted delivery to hepatosplenic tissues. This therapy also resulted in high BiTE concentrations, prolonged half-life, and robust antitumor efficacy against hematologic malignancies and melanoma [47]. This mRNA-based approach offers a flexible and cost-effective alternative for conventional recombinant antibody manufacturing and has therefore prompted a surge of clinical applications in cancer treatment [47].

### mRNA cancer vaccines encoding ILs and cytokines

Cytokines are among the most important agents in the immunotherapeutic treatment of cancer. In 1986 the FDA first approved the delivery of IFN-α for treatment of hairy cell leukemia [48], which was followed by approval of IL-2 in 1992 and 1998 for metastatic renal cancer and advanced melanoma, respectively [49, 50]. Currently, clinical studies are being conducted on a variety of cytokines for the treatment of various malignancies. However, these efforts faced tight therapeutic margins owing to their paracrine or autocrine effect and generally short half-life. *In vivo* studies of IL-12, IL-15, and IL-27 have frequently shown anticancer effectiveness [51], but to achieve the desired therapeutic window through systemic administration in the tumor microenvironment, higher concentrations of cytokines are required, which often results in dose-limiting toxicities [52, 53]. Alternative approaches with more efficient encapsulation, more proficient delivery of immunostimulatory cytokines to tumors, and lower systemic toxicity are urgently needed for clinical applications [54].

Encapsulated mRNA-encoding cytokines in LNPs have been shown to induce robust tumor infiltration of immune effectors and have inhibited tumor growth with reduced toxicity [54–56]. In comparison to carrier-free IL-12 or layer-free liposomal NPs, IL-12 therapies utilizing systemically delivered layer-by-layer NPs have exhibited diminished toxicity and sustained anti-tumor efficacy, resulting in a 30% complete survival rate in ovarian cancer [57]. mRNA-2416 LNPs by Moderna, which encapsulate mRNA-encoding wild type human OX40L (a ligand of OX40), are under clinical evaluation as a monotherapy and in combination with administered fixed doses of Durvalumab for the treatment of patients with ovarian cancer (NCT03323398). The intratumoral administration of mRNA 2416 in this trial was designed for up to 12 doses every 2 weeks, with four dose levels from 1 to 8 mg. mRNA-2416 was generally found to be well-tolerated at the different dose levels. The analysis of paired biopsies from injected lesions revealed an elevation in OX40L expression and PD-L1, as well as heightened T cell levels and pro-inflammatory activity. However, this trial was prematurely ended in July 2022 since the effectiveness objectives of neither the monotherapy nor combination treatment were fulfilled [24].

Similarly, mRNA 2752 LNPs, which encapsulate mRNA encoding human OX40L, IL-23, and IL-36γ, are now in clinical trials (NCTO3739931) for intratumoral injection alone and in combination with immune CPIs. mRNA-2752 was intratumorally administered every 2 weeks for up to seven doses, alone or in combination with the infusion of Durvalumab. A total of 23 solid tumor patients (monotherapy: *n* = 14; combination: *n* = 9) tolerated the treatment well with no dose-limiting toxicities. Six patients had stable disease, 10 had partial disease and 1 had partial responses (52% tumor reduction). A total of 5 individuals had tumor reduction in treated or untreated areas throughout treatment [25]. In recent years, cancer immunotherapy research has focused on IL-2 as a target to reduce regulatory T cell development, despite the challenge of potentially severe systemic toxicity. The peritumoral injection of ‘BALLkine-2’, recombinant human IL-2 (rIL-2) loaded in mesoporous silica NPs, elicited beneficial immunotherapeutic effects while mitigating concerns and adverse effects associated with rIL-2-related systemic and vascular toxicity. The improved therapeutic outcomes associated with BALLkine-2 were ascribed to two factors: the establishment of an rIL-2 depot facilitating the continuous release of rIL-2 and the heightened exposure of tumors to rIL-2, as well as the instantaneous localization of BALLkine-2 to secondary lymph nodes by DCs from the depot. These characteristics of locally injected BALLkine-2 make it attractive as an innovative cytokine therapy that offers lower medical cost and improved patient compliance [58].

### mRNA cancer vaccines encoding TAAs or TSAs

Cancer vaccines that target TAAs or TSAs can specifically attack and destroy malignant cells overexpressing the antigens of interest and can induce long-term therapeutic response through immunologic memory [1]. TAAs are attractive targets but are more suitable for certain solid tumors, such as melanoma and NSCLC. A typical example of the application of TAA targeting is the BNT111 cancer vaccine, the first candidate from the BioNTech FixVac platform to be tested in humans. BNT111 was FDA approved in 2021 for a Phase I trial (Lipo-MERIT, NCT02410733) to evaluate the safety and tolerability of vaccinating patients with stage III B, C, and stage IV melanoma. BNT111 is a nanoparticulate liposomal RNA (RNA-LPX) vaccine delivered intravenously that targets DCs in lymphoid compartments throughout the body. During the clinical study, the following TAAs for melanoma were utilized: New York esophageal squamous cell carcinoma 1 (NY-ESO-1), melanoma-associated antigen A3 (MAGE-A3), tyrosinase, and transmembrane phosphatase with tensin homology (TPTE). The study showed that T cells induced by FixVac were fully functional, recognized their target epitopes on melanoma cells, and exhibited strong cytotoxic activity. Although the vaccination proved effective as a single agent, the immunotherapy was markedly more effective in combination with anti-PD1 antibodies in CPI-positive tumor patients. Vaccine-induced T cells were of the PD1^+^ effector memory phenotype and therefore could be activated by the anti-PD1 antibodies. In pretreated, CPI-experienced patients with melanoma, the FixVac/anti-PD1 combo induced >35% tumor shrinkage. In individuals with CPI-naive metastatic melanoma, the objective response rates were comparable to PD1 blocking alone, as PD1 blockade works through the expansion of pre-existing antigen-specific T cells, many of which target mutation-derived neoantigens. Anti-PD1 treatment alone imposes a higher risk of disease recurrence because most patients have moderate to low mutational burden, which has been linked to a lower chance of pre-formed neoantigen-specific T cells. The four TAAs targeted are highly prevalent in human melanoma but are also found in normal cells. As vaccinations may induce both central and peripheral immune tolerance responses, their therapeutic effectiveness may be reduced. Consequently, most vaccines expressing TAAs are administered in conjunction with immunological CPIs. This Phase I study was completed in 2023 and Lipo-MERIT vaccine was found to display several anti-tumor activities that contributed to its therapeutic effect [27].

Two antigen-targeting vaccines, CV9201 [28] (NCT00923312) and CV9202 [29,30] (NCT01915524, NCT03164772) have been developed for NSCLC. CV9201 is an RNActive^®^-based cancer immunotherapy encoding five NSCLC-antigens: NY-ESO-1, melanoma antigen family C1/C2, survivin, and the oncofetal antigen 5T4. CV9202 (also called BI1361849) is an active cancer immunotherapeutic comprising protamine-formulated, sequence-optimized mRNA encoding six NSCLC-associated antigens (NY-ESO-1, MAGE-C1, MAGE-C2, survivin, 5T4, and MUC-1). Clinical trials of the two vaccines have shown tremendous tolerance and improved immune response following treatment. These findings have encouraged the clinical development of mRNA-based immunotherapy for NSCLC, especially in combination with CTLA-4 [28–31].

### mRNA cancer vaccines encoding neoantigens and personalized vaccines

Mutated, non-self-peptides generated in cancer cells due to non-synonymous mutations are processed and presented by MHC on the cell surface, proficiently stimulating T-cell responses. These antigens, known as neoantigens, are capable of potent immunogenicity, arise from somatic mutations typically not exhibited in normal cells, and can be classified into two categories: private and public neoantigens. Private neoantigens differ from patient to patient and are designed as custom-made therapies based on the specifications of each patient [59]. One previous clinical study demonstrated that 62 of 75 (83%) patients with common gastrointestinal cancers released tumor-infiltrating lymphocytes (TIL) that recognized neoantigens, and that the majority of the neoantigen determinants were unique and not shared among patients [60]. This type of cancer vaccination permits individualized diagnosis and therapy for each patient and has become the focus of most current clinical studies. A Phase I (NCT03313778) open-label, multicenter research trial examining the safety, tolerability, and immunogenicity of the personalized vaccine mRNA-4157 (V940) alone and in combination with a CPI (Pembrolizumab) in patients with resected solid tumors found that mRNA-4157 (V940) was safe and well-tolerated at all dose levels tested. Clinical responses have been observed in combination with Pembrolizumab and neoantigen-specific T cells have been induced, supporting the advancement of mRNA-4157 to Phase II [32]. So far, 3-year data from a Phase II (NCT03897881) trial of mRNA-4157 (V940) in combination with Pembrolizumab has demonstrated sustained improvement in recurrence-free survival [33] and distant metastasis-free survival [34] versus Pembrolizumab alone in patients with high-risk stage III/IV melanoma following complete resection. Multiple Phase III trials of mRNA-4157 (V940) plus Pembrolizumab versus Pembrolizumab (NCT06077760, NCT06295809, NCT05933577) were subsequently initiated. Similarly, the BioNTech vaccine RO7198457 (BNT122) is currently being evaluated in a multi-step, open-label, phase II (NCT04486378) randomized trial versus watchful waiting in patients with circulating tumor DNA (ctDNA)-positive, surgically resected Stage II/III rectal cancer or Stage II (high risk)/Stage III colon cancer [37].

Mutated antigens that are conserved among cancer patients are considered public neoantigens for groups with analogous genetic alterations. The high specificity of neoantigens to cancer cells due to underlying mutations, while exerting minimal toxicity to non-cancerous cells, propels different screening techniques in cancer immunotherapy. Several strategies to screen for candidate neoantigens, such as next-generation sequencing technology, whole-exome sequencing, computer algorithms, and immunological effects evaluation, are available to facilitate more precisely identification and destruction of cancer cells by the immune system [61]. However, the current algorithm utilized for predicting neoantigens has a few drawbacks, since it is largely confined to *in vitro* binding affinity data and is computationally constrained. As a result, it generates unavoidably large levels of false positives. Hao *et al*. [62] have therefore proposed a deep convolutional neural network titled APPM (antigen presentation prediction model) to predict antigen presentation in the context of HLA class I alleles. The APPM prediction, combined with the immune epitope database, can improve the accurate prediction of neoantigens. Another model, dubbed EDGE, was previously developed by Bulik-Sullivan *et al*. [63] based on a large HLA peptide and genomic dataset from various human tumors and improved the accuracy of HLA antigen prediction by as much as 9-fold.

### mRNA cancer vaccines improving CAR T-cell therapy

Adoptive T-cell therapy is an umbrella term for therapeutic treatments involving the administration of enhanced or altered autologous cancer-specific T cells. This involves the *ex vivo* modification of isolated autologous cells to enhance patients’ T-cells to identify and attack treatment-resistant cancer cells [64]. There are two main types of T-cell therapy: TIL therapy and CAR-modified T-cell therapy. The lymphocyte treatment was deemed ineffective due to its inability to eradicate the tumor or counteract the signals that the tumor emits to inhibit the immune system [65], and therefore recent attention has focused on development of CAR T-cell therapy as an advanced personalized cancer immunotherapy. This engineered T-cell therapy has received approval from the FDA and the European Medicine Agency for clinical application in hematological cancers, including acute lymphoblastic leukemia and diffuse large B-cell lymphoma [66, 67]. CAR sequencing represents a major modification to T cells, and transduction is achieved by the use of retro- or lenti-viral transduction, which has raised concerns regarding risks of insertional mutagenesis, immunogenicity, and limited payload capacity [68]. In response to these concerns, mRNA technology now enables transient expression of CAR, since mRNA sequencing can be customized, and the molecules are subject to decay after translation, averting any risk of genomic vector integration [69]. As an alternative to integrating viral vectors, electroporation (EP) of CAS9 mRNA into human T cells allows directed integration of a CD19-specific CAR to the T cell receptor α constant (TRAC) locus, resulting in constant CAR expression as well as improved T cell potency [70]. The advantages of TRAC include promoting optimal baseline expression that can eradicate CAR tonic signaling in the absence of antigen and permit effective CAR internalization upon single or multiple contacts with antigen. TRAC also produces a balanced transcriptional response, resulting in kinetically optimal recovery of baseline CAR expression after antigen engagement. In contrast to T-cells with higher CAR expression, the *TRAC*-CAR profile has exhibited superior tumor eradication via decreased T-cell differentiation and exhaustion [71, 72]. However, EP uses pulsed electric fields, which entail a risk of cytotoxicity and irreversible loss of cytoplasmic content, resulting in failure to guarantee consistent membrane penetration across cells for delivery [73]. Thus, further investigation is necessary to analyze the potential risks associated with the long-term expression of transgenes and their behavior in cells [74]. A recent investigation into the use of LNP to deliver CAR-mRNA to T-cells *ex vivo* also revealed that its efficacy was prolonged in comparison to EP. Additional findings indicated that EP-CAR T cells exhibited decreased viability and efficiency against target cells. This also increased fatigue and heightened off-target toxicity compared to LNP-CAR T cells 1 day post-transfection [75]. Since the end of the last decade, nanoparticles, especially LNPs, have come to dominate non-viral delivery systems for lymphocyte transfection. The exploitation of LNPs has been shown to facilitate the delivery of CAR-encoding mRNA into primary human T-cells, resulting in functional CAR T-cells that engage in high levels of tumor-killing activity [76–78].

## Conclusions and future perspectives

mRNA-based therapeutics are now the foundation of many new treatments for various human malignancies and have transformed gene editing, protein replacement therapies, cell reprogramming, and immunotherapies via their immense applications. As mRNA is synthesized through cell-free methods, the quick and cost-effective production of mRNA-based medicines is feasible and efficient. However, the application of these therapies is complicated by the inherent chemical instability of mRNA and its susceptibility to hydrolysis catalyzed by nucleases. Although mRNA vaccinations proved critical in combating the COVID-19 pandemic, several significant obstacles must be overcome for mRNA technology to succeed in treating diseases such as cancer. A key obstacle is the lack of practical and scalable mRNA synthesis techniques that are compatible with current pharmaceutical technology. Clinically translatable mRNA transporters with both appropriate stability in the systemic circulation and resolution of current immunogenicity issues are required for the development of potent mRNA therapies. Recent advances in non-viral delivery systems and the development of novel, effective, transfecting nanomaterials have provided solutions to some of these challenges. Several anti-cancer immunotherapeutic techniques, including therapy vaccines, monoclonal antibodies, and CAR cells combined with mRNA-based technologies through LNPs, have likewise enhanced the efficacy of various treatments. More studies are needed to determine the causes of the low levels of mRNA transfection effectiveness shown by non-viral vehicles, particularly in lymphocytes, monocytes, and other cells that have proved difficult to transfect. Combining mRNA-based therapies with other cancer treatments, including CPIs, chemotherapy, and radiation, may represent a viable strategy for enhancing the effectiveness of these therapies. To increase delivery, the gaps in efficiency and usefulness found between small animals (e.g. mice, rats), larger animals (non-human primates), and humans must be bridged by expediting the nanoparticle discovery pathway. It will be exciting to see the results of the current wave of clinical vaccination studies. These outcomes will have a significant impact on the whole arena of mRNA-based therapeutics as well as the future landscape of biomedicine.
